# Enhancement of Gap Junction Function During Acute Myocardial Infarction Modifies Healing and Reduces Late Ventricular Arrhythmia Susceptibility

**DOI:** 10.1016/j.jacep.2016.03.007

**Published:** 2016-10

**Authors:** Fu Siong Ng, Jeremy M. Kalindjian, Simon A. Cooper, Rasheda A. Chowdhury, Pravina M. Patel, Emmanuel Dupont, Alexander R. Lyon, Nicholas S. Peters

**Affiliations:** Imperial College, London, United Kingdom; Myocardial Function, National Heart & Lung Institute, Imperial College, London, United Kingdom

**Keywords:** electrophysiology, fibrosis, gap junctions, myocardial infarction, ventricular arrhythmia, CV, conduction velocity, Cx43, connexin43, GJ, gap junction, IBZ, infarct border zone, MI, myocardial infarction, PBS, phosphate-buffered saline, PES, programmed electrical stimulation, VT, ventricular tachycardia

## Abstract

**Objectives:**

The purpose of this study was to investigate the effects of enhancing gap junction (GJ) coupling during acute myocardial infarction (MI) on the healed infarct scar morphology and late post-MI arrhythmia susceptibility.

**Background:**

Increased heterogeneity of myocardial scarring after MI is associated with greater arrhythmia susceptibility. We hypothesized that short-term enhancement of GJ coupling during acute MI can produce more homogeneous infarct scars, reducing late susceptibility to post-MI arrhythmias.

**Methods:**

Following arrhythmic characterization of a rat 4-week post-MI model (n = 24), another 27 Sprague-Dawley rats were randomized to receive rotigaptide to enhance GJ coupling (n = 13) or to saline control (n = 14) by osmotic minipump immediately prior to and for the first 7 days following surgically induced MI. At 4 weeks post-MI, hearts were explanted for ex vivo programmed electrical stimulation (PES) and optical mapping. Heterogeneity of infarct border zone (IBZ) scarring was quantified by histomorphometry.

**Results:**

Despite no detectable differences in infarct size at 4 weeks post-MI, rotigaptide-treated hearts had reduced arrhythmia susceptibility during PES (inducibility score for rotigaptide: 2.4 ± 0.8; for control: 5.0 ± 0.6; p = 0.02) and less heterogeneous IBZ scarring (dispersion of IBZ complexity score: rotigaptide: 1.1 ± 0.1; control: 1.4 ± 0.1; p = 0.04), associated with an improvement in IBZ conduction velocity (rotigaptide: 43.1 ± 3.4 cm/s; control: 34.8 ± 2.0 cm/s; p = 0.04).

**Conclusions:**

Enhancement of GJ coupling for only 7 days at the time of acute MI produced more homogeneous IBZ scarring and reduced arrhythmia susceptibility at 4 weeks post-MI. Short-term GJ modulation at the time of MI may represent a novel treatment strategy to modify the healed infarct scar morphology and reduce late post-MI arrhythmic risk.

## Graphical abstract

**Figure:**
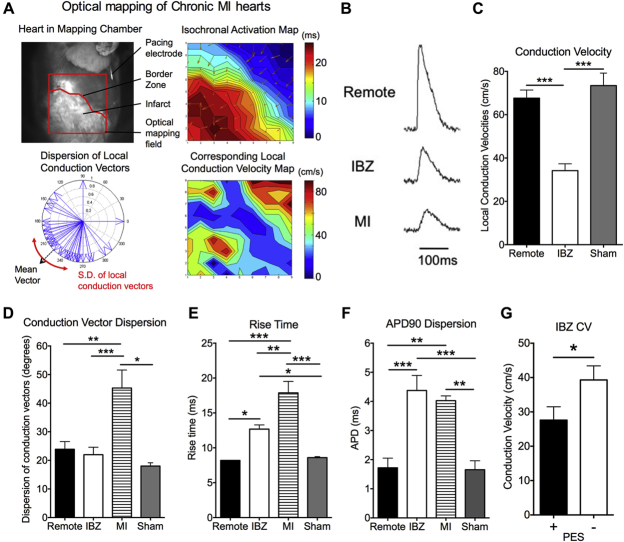

Ventricular arrhythmia episodes are responsible for most of the 300,000 annual sudden cardiac deaths in the United States (1), with myocardial infarction (MI) being the principal underlying cause. Many sudden deaths in patients with previous MI occur months to years after their index event. Heterogeneity of infarct scarring has been identified as the determinant of late post-MI arrhythmia, with increased heterogeneity of fibrosis being associated with increased arrhythmic risk 1, 2, 3. In the infarct border zone (IBZ), heterogeneous scarring produces bundles of surviving myocardium within areas of dense fibrotic scar, creating the substrate for re-entrant circuits causing late post-MI ventricular tachycardia (VT) 4, 5, 6. Therapeutic strategies to homogenize infarct scarring, both by ablation or pharmacologically, have been shown to be antiarrhythmic in the chronically infarcted heart 7, 8, 9.

Gap junctions (GJs) are clusters of transmembrane channels that mediate coupling of the cytoplasmic compartments of adjacent cells and allow cell-to-cell transfer of ions and small molecules. Studies have shown that modulating GJ coupling can modify intercellular passage of products of cell necrosis, affect infarct spread, and may have small effects on the size of the healed infarct 10, 11, 12. Enhancing GJ coupling during MI at a time when natural GJ uncoupling occurs (13) would be expected to increase gap junctional exchange of chemical mediators of cell death and survival between healthy and dying cells at the ischemic border, thus homogenizing the distribution of cell death and survival during MI. Although any resulting myocardial salvage may be inadequate to significantly alter myocardial mechanical function, even subtle alterations of the morphology of the scar border relating to increased homogeneity of scarring in the healed infarct may have important effects on late post-MI arrhythmia susceptibility.

We hypothesized that enhancing GJ coupling only for a limited duration at the time of MI, could reduce late arrhythmia susceptibility in a chronically infarcted heart, resulting from greater homogeneity of scarring in the healed infarct. We characterized the arrhythmic behavior and electrophysiology in a rat model of healed MI and investigated the effects of short-term GJ enhancement during acute MI on the morphology and arrhythmia susceptibility of the healed infarct scar.

## Methods

Methods are described briefly here. For full details, please see the Online Appendix.

### Ethical approval

This work was performed in accordance with standards set out in the United Kingdom Animals (Scientific Procedures) Act 1986 and was approved by Imperial College London Ethical Review Board and carried out under Project License PPL 70/7033.

### Experimental protocols

To characterize the arrhythmic behavior and electrophysiology of our 4-week chronic MI model, 24 male Sprague-Dawley rats (250 to 300 g) were subjected to surgical MI by left anterior descending (LAD) artery ligation as previously described (14), while 4 rats underwent sham MI surgery. After 4 weeks of healing, rats were sacrificed, and hearts were explanted, perfused ex vivo, and subjected to optical mapping of transmembrane voltage as previously described (15) and to programmed electrical stimulation (PES) to provoke ventricular arrhythmia.

To assess the effects of short-term GJ modulation during acute MI on the healed infarct morphology and arrhythmia susceptibility at the chronic healed MI phase, another 27 rats were randomly allocated to 1 of 2 groups receiving 7 days of either rotigaptide to enhance GJ coupling (n = 13) or phosphate-buffered saline (PBS) as the control group (n = 14). We had previously confirmed that rotigaptide enhances GJ coupling in ventricular myocardium in the context of acute ischemia/metabolic stress in separate immunoblotting and ex vivo optical mapping experiments (described in Online Appendix), consistent with previously published studies (16).

Animals were given a bolus of GJ modulator or vehicle subcutaneously immediately before LAD ligation (2.5 nmol/kg rotigaptide or 0.5 ml of PBS). GJ modulator or vehicle was then delivered for the first 7 days post-MI, using intraperitoneal osmotic minipumps (infusion rate of rotigaptide: 0.11 nmol/kg/min; or PBS: 2 ml/week) (10). At 4 weeks post-MI (i.e., 3 weeks after discontinuation of rotigaptide administration), hearts were explanted for ex vivo optical mapping with arrhythmia provocation studies (PES). The vulnerability of hearts to PES-induced arrhythmias was quantified using a previously described and previously validated arrhythmia inducibility score for PES in rat hearts (17). Hearts were then frozen and sectioned for histological staining with Masson’s trichrome for maximum contrast and differentiation between scar tissue and surviving myocardium and for connexin43 (Cx43) immunolabeling.

### Histology and histomorphometry

Infarct size was quantified by planimetry using previously validated methods 18, 19. Briefly, endocardial and epicardial circumferences of the infarct were measured for each section, and the infarct size was quantified as the proportion of endocardial and epicardial circumferences bounded by the transmural infarct.

The complexity of IBZ scarring and degree of heterogeneity of fibrosis was quantified using an interface complexity ratio (ICR), defined as the ratio of the length of interface between fibrosis and surviving myocardium to the area of fibrosis in that microscopic field (Online Appendix, Online Figure 2). IBZs with greater heterogeneity of fibrosis have greater ratios, that is, greater interface between fibrotic and myocardial tissue per unit area of fibrosis. The interobserver and intraobserver coefficients of variation for this method were 12% and 10%, respectively. For each heart, 10-μm slices were taken at 500-μm intervals across the entire infarct for staining with 27 ± 5 IBZ microscopic fields analyzed per slice. ICR values were then averaged to give a single mean value and a single SD value, as a measure of dispersion, per heart. All experiments and analyses were performed blinded to treatment group.

### Data analysis and statistics

Optical mapping data were analyzed as previously described 15, 20, 21. Activation maps were generated, and local conduction velocities and vectors were derived using MATLAB R2010a software (MathWorks, Marlborough, Massachusetts). Analysis of variance tests were performed to compare means between multiple groups, and post hoc Tukey test was used if analysis of variance was significant. Student *t* tests were used to compare means between 2 groups. A p value of <0.05 was considered significant. All values are mean ± SEM.

## Results

### Characterization of conduction, optical action potentials, and arrhythmogenesis in the chronic healed MI model

**Figure 1:**
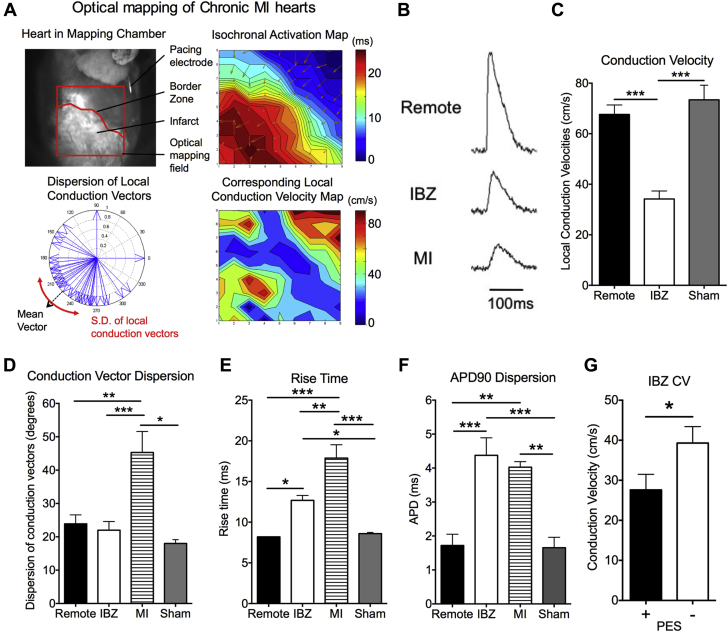
Electrophysiological and Arrhythmia Determinants of Chronic MI Model **(A)** Optical mapping of chronic MI hearts. **(B)** Representative optical action potentials from remote myocardium, IBZ, and infarct zone. **(C)** Slower CVs at IBZ compared with remote myocardium. **(D)** Conduction vector angles were more heterogeneous in the infarct zone (MI). **(E)** Prolonged rise times at the infarct zone and IBZ. **(F)** Increased dispersion of optical APDs at the infarct zone and IBZ. **(C to F)** n = 16 for MI hearts, and n = 4 for sham hearts. **(G)** IBZ CV was slower in PES(+) hearts (n = 8) than in PES(−) hearts (n = 8). *p < 0.05; **p < 0.01; ***p < 0.001. APD = action potential duration; CV = conduction velocity; IBZ = infarct border zone; PES = programmed electrical stimulation.

Sixteen of 24 infarcted rats (67%) and all 4 sham-operated rats survived the acute surgery. Optical mapping studies were performed at 4 weeks post-MI. Figure 1A shows representative activation maps and local conduction velocity (CV) maps for a chronically infarcted hearts, and Figure 1B shows representative optical action potentials from the remote noninfarcted myocardium, the IBZ, and the infarct zone. There was a 49% reduction in IBZ CV compared with remote noninfarcted myocardium (34.1 ± 3.2 cm/s vs. 67.6 ± 3.8 cm/s, respectively; p < 0.0001) (Figure 1C). CVs in the remote myocardium of MI hearts were not different from the same myocardial region of sham-operated hearts (73.4 ± 5.8 cm/s). There was increased dispersion of conduction vector angles in the infarct zone compared with that in the IBZ and remote myocardium, demonstrating greater heterogeneity in directions of activation within the infarct (Figure 1D).

Optical action potential rise times in the infarct zone and IBZ were prolonged compared with those recorded at the remote, viable myocardium and those from sham-operated hearts (Figures 1E). Mean action potential durations were not different among the infarct zone, the IBZ and remote myocardium, but there was greater spatial variability of action potential durations in the infarct zone and IBZ than in remote myocardium and in sham-operated hearts (Figure 1F).

Hearts were classified for arrhythmia susceptibility based on the PES experiments. All hearts were ranked by arrhythmia susceptibility and then divided into 2 groups based on the median values. Hearts in the more arrhythmic (+) group had values above the median, and hearts in the less arrhythmic (−) group had values below the median. As shown in Figure 1G, IBZ CVs were significantly slower in the PES(+) hearts than in the PES(−) hearts (27.6 ± 3.8 cm/s vs. 39.3 ± 4.1 cm/s, respectively; p = 0.04), suggesting that IBZ CV is a determinant of susceptibility to ventricular arrhythmias on PES in chronic MI hearts.

### Effects of rotigaptide treatment on conduction, optical action potentials, and arrhythmogenesis in healed MI

**Figure 2:**
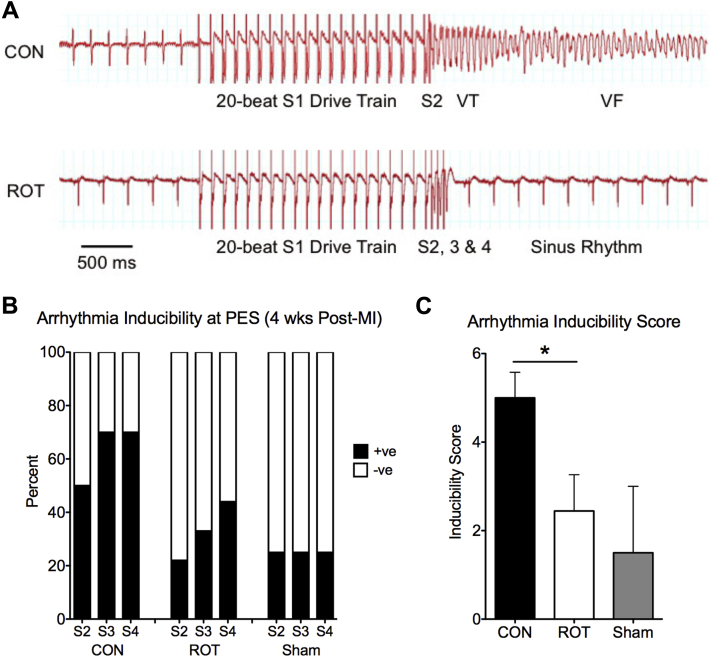
Reduced Arrhythmia Susceptibility on PES in Rotigaptide-Treated Hearts at 4 Weeks Post-MI **(A, Top)** Example of VT/VF induced in a control (CON) heart during PES. **(A, Bottom)** Example of PES in a rotigaptide (ROT) heart, with no arrhythmias induced with 3 extrastimuli. **(B)** Proportions of hearts with VT/VF (duration >1 s) induced with PES. **(C)** Reduced arrhythmia inducibility scores for rotigaptide hearts (n = 9) compared with that of control MI hearts (n = 10) (*p < 0.05). Data from sham-operated hearts (n = 4) presented for comparison. +ve = PES positive; −ve = PES negative; MI = myocardial infarction; VT/VF = ventricular tachycardia/ventricular fibrillation; other abbreviations as in Figure 1.

Of the animals randomized to treatment with either rotigaptide (n = 13) or control (n = 14), 9 animals from the rotigaptide group and 10 from the control group survived acute MI surgery (acute mortality in rotigaptide animals: 31%; 29% in control; p = NS). At 4 weeks post-MI, hearts of animals treated with rotigaptide for the first 7 days post-MI had reduced arrhythmia inducibility at PES than controls, with VT/ventricular fibrillation (VF) induced in fewer rotigaptide-treated hearts for any given number of extrastimuli (Figures 2A and 2B) and a reduction in the arrhythmia inducibility score (rotigaptide: 2.4 ± 0.8; control: 5.0 ± 0.6; p = 0.02) (Figure 2C). These findings indicate a difference in substrate in rotigaptide hearts compared with that in controls, which rendered hearts more resistant to PES-induced ventricular arrhythmia.

**Figure 3:**
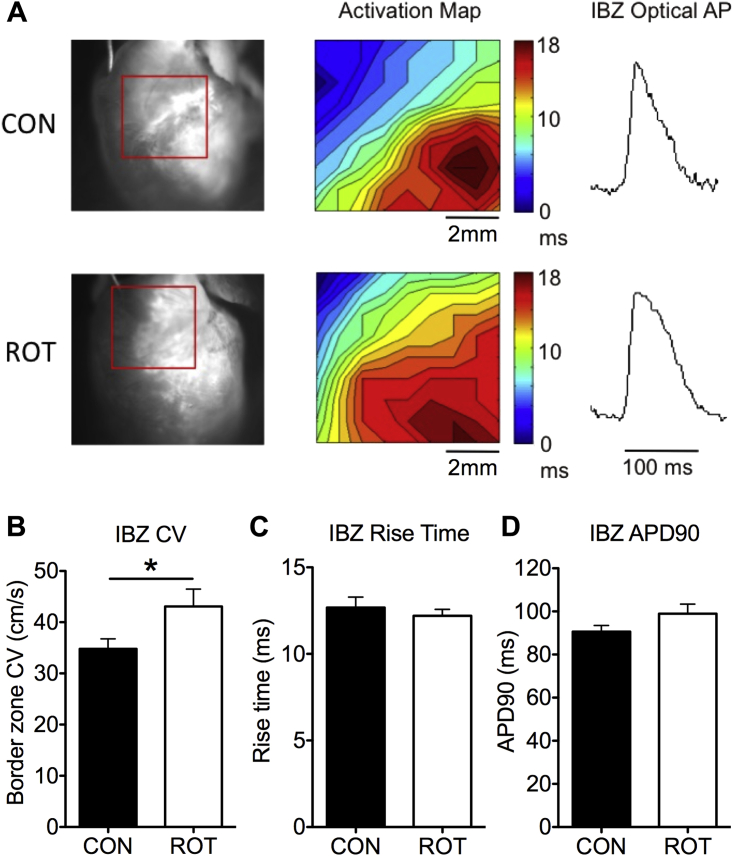
Improvement in IBZ CV in Rotigaptide-Treated Hearts **(A)** Representative activation maps show conduction slowing at the IBZ of control and rotigaptide hearts and representative IBZ optical APs. **(B)** Increase in IBZ CV in rotigaptide-treated hearts (n = 9) compared with untreated control hearts (n = 26). **(C and D)** No differences in optical AP rise times and AP duration between groups.

Figure 3A shows representative activation maps and optical action potentials for control and rotigaptide hearts. Consistent with the demonstration that IBZ CV is a determinant of arrhythmia susceptibility, there was a 24% increase in IBZ CV in rotigaptide-treated hearts compared with untreated post-MI animals (rotigaptide: 43.1 ± 3.4 cm/s; control: 34.8 ± 2.0 cm/s; p = 0.04) (Figure 3B). There were no differences in optical action potential rise times and durations between groups (Figures 3C and 3D).

### Rotigaptide treatment did not alter infarct size

**Figure 4:**
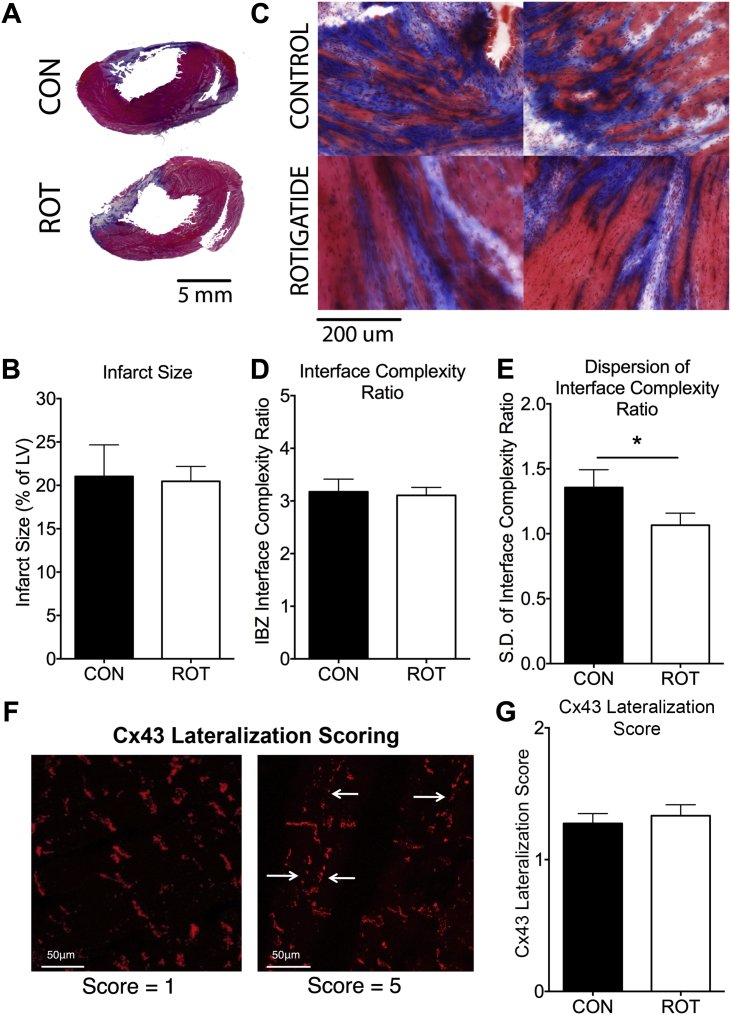
Reduced Heterogeneity of Fibrosis at IBZ of Rotigaptide-Treated Hearts **(A)** Representative biventricular slices from the mid-ventricles of MI hearts stained with Masson’s trichrome. **(B)** No differences are seen in infarct size between groups. **(C)** Sample images of the IBZ from control and rotigaptide hearts show complex interaction between fibrosis **(blue)** and surviving myocardium **(reddish-pink)**. **(D)** Mean IBZ ICRs were not different between groups. **(E)** Rotigaptide reduced the dispersion of ICR (SD of ICR values within each heart) compared to control MI hearts (*p < 0.05). **(F)** Cx43 lateralization scoring system. **(Left)** Normal Cx43 localization at the intercalated discs. **(Right)** Significant lateralization of Cx43 as shown by **arrows**. **(G)** Cx43 lateralization scores were not different between groups. CON n = 10; ROT n = 9. ICR = interface complexity ratio.

Surgical LAD artery ligation produced transmural infarcts with compensatory hypertrophy of noninfarcted myocardium, as shown using Masson’s trichrome-stained biventricular sections in Figure 4A. Infarct sizes by planimetry were not different between groups (control: 21.0 ± 3.6%; rotigaptide: 20.5 ± 1.7%; p = NS) (Figure 4B), suggesting that acute GJ enhancement during MI did not grossly alter infarct size and that this could not account for the reduced susceptibility to PES arrhythmia in rotigaptide hearts.

### Rotigaptide reduced heterogeneity of fibrosis at the IBZ

Differences in IBZ morphology and structural heterogeneity were determined using the interface complexity ratio (ICR), a measurement of fibrosis complexity at the IBZ, with greater ratios representing more complex morphologies (Online Appendix). Figure 4C shows sample images of IBZ from control and rotigaptide hearts. Although mean interface complexity ratios were not significantly different between groups (control: 3.2 ± 0.2; rotigaptide: 3.3 ± 0.2; p = NS) (Figure 4D), the degree of heterogeneity of IBZ scarring was reduced after rotigaptide treatment (SD of ICR values within each heart, control: 1.4 ± 0.1; rotigaptide: 1.1 ± 0.1; p = 0.04) (Figure 4E). The reduction in ICR variability within each heart for the rotigaptide group points toward more homogeneous patterns of IBZ scarring, whereas control hearts exhibited a greater range of IBZ scar morphologies within each heart.

### Rotigaptide did not alter post-MI Cx43 maldistribution

There were no differences in mean Cx43 lateralization scores between treatment groups (control: 1.3 ± 0.1, rotigaptide: 1.3 ± 0.1; p = NS) (Figures 4F and 4G) or in variability of the Cx43 lateralization scores (SD of Cx lateralization score within each heart for control: 0.5 ± 0.1; and for rotigaptide: 0.5 ± 0.1; p = NS).

## Discussion

The principal and important finding of this study is the proof of concept of a highly novel antiarrhythmic strategy of modifying infarct healing by short-term enhancement of GJ function during acute MI, which modifies the healed arrhythmogenic substrate by reducing inhomogeneities of fibrosis at the healed IBZ without gross changes in infarct size, thus reducing VT/VF inducibility late post-MI. The homogenization of scarring was associated with a corresponding improvement in macroscopic CV across the IBZ.

It is important to emphasize the distinction from previous GJ enhancement studies focused on the direct acute electrophysiological effects of rotigaptide on conduction (22), rather than this paradigm shift of modifying the molecular biology of the disease process itself with an enduring antiarrhythmic effect on infarct scar morphology and a reduction in arrhythmia susceptibility 3 weeks after discontinuation of rotigaptide.

### Enhancement of GJ coupling reduced heterogeneity of scarring and fibrosis at the healed IBZ

GJ channels are known to mediate the spread of small molecules of <1 kDa in molecular weight, including the passage of mediators of cell death and cell survival during MI (23). During acute MI, closure of GJ channels occurs (13), thus preventing the passage of these molecules between cells and enhancing differential survival between adjacent cells and clusters of cells because of the heterogeneities in local vascular supply, coronary blood flow, and cellular metabolism 24, 25, thus leading to heterogeneous cell death. In keeping with this concept, our principal histomorphological finding of homogenization of scarring at the IBZ with short-term rotigaptide treatment, as supported by the reduced dispersion of ICR values for each heart, is consistent with possible enhanced gap-junctional exchange of chemical mediators of cell death and survival between healthy and dying cells of the IBZ resulting in more homogeneous patterns of cell death and infarction (23) (Online Figure 6). Potential mediators of cell death that can pass through GJ channels include Ca^2+^, inositol triphosphate (IP3), cyclic adenosine monophosphate, and cyclic guanosine monophosphate (26), whereas potential “rescue messengers” that can protect from cell death include ascorbic acid, reduced glutathione, glucose, and adenosine triphosphate (27).

### Enhancement of GJ coupling during acute MI reduced late post-MI arrhythmia susceptibility

The reduction in heterogeneity of patterns of fibrosis and scarring at the healed IBZ of the rotigaptide-treated hearts was associated with a reduction in susceptibility to ventricular arrhythmias on PES at 4 weeks post-MI. The observed reduction in heterogeneity of IBZ scarring would be expected to reduce the occurrence of adjacent areas of fast and slow conduction and, therefore, reduce the likelihood of arrhythmia.

Our finding is consistent with delayed-enhancement cardiac magnetic resonance studies, which have found that increased scar heterogeneity correlated strongly with inducibility of monomorphic VT (2) and predicted post-MI mortality (3), with the zones of greatest tissue heterogeneity shown to contain critical isthmus sites of scar-related VT (28). Our proposed strategy for reducing IBZ scar heterogeneity parallels the interventional approach of substrate modification by catheter ablation, which has the effect of homogenizing the infarct scar, thereby reducing or abolishing overall arrhythmia burden 7, 9. Recent clinical studies have demonstrated that extensive ablation using a combined endocardial and epicardial approach to homogenize infarct scars can improve freedom from arrhythmia (7), whereas the similar but less extensive ablation approach of homogenizing scar tissue by ablating conducting channels has also been shown to reduce VT recurrence (9). Our strategy is also supported by recent experiments demonstrating that the homogenization of ventricular scar by the application of collagenase can create a less arrhythmic substrate (8).

### Improvement in conduction velocity at the healed IBZ of rotigaptide-treated hearts

Improvement in macroscopic CV across the IBZ in rotigaptide-treated hearts is consistent with the finding of reduced scar heterogeneity in those hearts, which would be expected to reduce the tortuosity and conduction path lengths across the IBZ, as described above (5). These findings further support a central role for the IBZ in post-MI arrhythmia and lend weight to the notion that treatments that alter IBZ scar morphology can alter post-MI arrhythmia susceptibility.

### Rotigaptide did not significantly alter infarct size

There were no gross differences in infarct size between control and rotigaptide. Previous studies looking specifically at the effects of GJ enhancement on infarct size have produced conflicting results, with a study demonstrating a minor increase in infarct size (11), whereas studies using the pharmacological GJ modulators rotigaptide and danegaptide have shown minor reductions in infarct size 10, 12. These disparities may reflect differences in animal models and of timing and duration of enhancement of coupling, as well as differences in methods of measuring infarct size. In any case, any differences in scar size are at most minimal, and although too small to significantly salvage mechanical contractile function, our findings indicate that even subtle scar homogenization significantly reduces arrhythmogenesis with the potential for clinical impact.

### Study limitations

Optical recordings of transmembrane potential were limited to a depth of several cells at the subepicardium, which meant we were unable to precisely map the location of re-entrant circuits of the induced arrhythmias and had to extrapolate the electrophysiology of deeper myocardial layers from subepicardial data, although the use of optical mapping to interrogate the electrophysiology of the IBZ has previously been validated (29).

Although it was not possible to measure directly the effects of rotigaptide on GJ coupling during acute MI in the in vivo cohort, we confirmed in parallel ex vivo studies that rotigaptide has the expected effects on conduction velocity and Cx43 phosphorylation consistent with GJ enhancement in acute ischemia and acute metabolic stress (Online Appendix).

## Conclusions

Enhancement of GJ coupling for a limited duration only during the acute phase of MI can reduce inhomogeneities of fibrosis in the healed IBZ while reducing late susceptibility to PES-induced ventricular tachyarrhythmias at the chronic healed infarct phase and may represent a novel clinically-applicable therapeutic strategy to reduce late post-MI ventricular arrhythmias.

Perspectives **COMPETENCY IN MEDICAL KNOWLEDGE:** Increased heterogeneity of myocardial scarring after MI is associated with greater arrhythmia susceptibility. Approaches to homogenize scar, such as ablation, have demonstrated anti-arrhythmic benefit. Here, we propose a novel pharmacological strategy to homogenize scar by peri-MI GJ enhancement.

**TRANSLATIONAL OUTLOOK:** Enhancement of GJ coupling during acute MI may represent a novel, clinically applicable therapeutic strategy to reduce heterogeneities of scarring at the IBZ and reduce post-MI ventricular arrhythmias. In our proof-of-concept study, a loading dose of rotigaptide was administered immediately pre-MI to allow for therapeutic concentrations at the time of MI. Further experiments are required to determine if short-term GJ enhancement commencing after MI onset or chronic GJ enhancement pre-MI confer similar beneficial effects before moving into clinical trials. Furthermore, studies to determine the safety and side-effect profile of limited-duration and chronic GJ enhancement in humans are also required prior to clinical translation.
