# Supplementary material for: Enhancement of Gap Junction Function During Acute Myocardial Infarction Modifies Healing and Reduces Late Ventricular Arrhythmia Susceptibility
Source: JACC Clin Electrophysiol. 2016 Oct;2(5):574–82. doi: 10.1016/j.jacep.2016.03.007 (PMC5076465; doi:10.1016/j.jacep.2016.03.007)

Supplementary Material for the following article:

TITLE: Enhancement of Gap Junction Coupling During Acute Myocardial Infarction Modifies Healing and Reduces Late Ventricular Arrhythmia Susceptibility

AUTHORS: Fu Siong Ng, MBBS, PhD, Jeremy M. Kalindjian, MBBS, Simon A. Cooper, MBBS, Rasheda A. Chowdhury, PhD, Pravina M. Patel, BSc, Emmanuel Dupont, PhD, Alexander R. Lyon, BMBCh, PhD, Nicholas S. Peters, MBBS, MD.

-

Supplementary Materials & Methods:

Ethical approval

This work was performed in accordance with standards set out in the United Kingdom Animals (Scientific Procedures) Act 1986, and conforms to Directive 2010/63/EU of the European Parliament. The work was approved by the Imperial College London Ethical Review Board and carried out under Project License PPL 70/7033.

Study protocol

The study protocol is summarized in **Supplementary Figure 1A**. Firstly, to investigate the arrhythmia determinants and the electrophysiology of our rat model of chronic myocardial infarction (MI), a cohort of 24 male Sprague-Dawley rats (250g) was subjected to surgical myocardial infarction by left anterior descending (LAD) artery ligation (1, 2), while another four rats underwent sham MI surgery where a ligature was placed around the LAD artery but not tightened. At chronic healed MI phase at four weeks post-myocardial infarction, rats were sacrificed, and hearts were

explanted, perfused *ex vivo* and subjected to optical mapping of transmembrane voltage, as described below. Hearts were also subjected to programmed electrical stimulation (PES) to provoke ventricular arrhythmias. Details of the arrhythmia provocation protocols are presented below.

Following characterisation of the arrhythmic behaviour and electrophysiology of our chronic MI model, another 27 Sprague-Dawley rats were subjected to surgical MI in order to assess the effects of short-term gap junction modulation during acute MI on the healed infarct morphology and late post-MI arrhythmia susceptibility. These animals were randomly allocated to one of 2 groups: (1) treatment with rotigaptide to enhance gap junction coupling (n=13), or (2) phosphate-buffered saline (PBS) as the control group (n=14), and were pre-treated with a bolus of gap junction modulator or vehicle subcutaneously immediately before LAD ligation (2.5nmol/kg rotigaptide or 0.5ml PBS). Gap junction modulator or vehicle was then delivered for 7 days post-MI using osmotic minipumps (Azlet 2ML1, Charles River, UK), which were placed in the peritoneal cavity at the time of the myocardial infarction surgery (Infusion dose: Rotigaptide 0.11nmol/kg/min, or PBS 2ml/week). Doses were selected based on the existing literature (3). At four weeks post-MI (3 weeks after discontinuation of rotigaptide administration), rats were sacrificed, and hearts were perfused *ex vivo* for optical mapping and programmed electrical stimulation (PES). Hearts were then frozen and sectioned for histological staining with Masson’s trichrome and for Cx43 immunolabeling. The complexity of infarct border zone (IBZ) scarring was quantified by histomorphometry. All experiments and analyses were performed blinded to treatment group.

Anaesthesia and pre-operative management

Each rat was first placed inside an induction chamber and anaesthetised with a mixture of 5% isoflurane and 95% oxygen. The rat was then weighed and transferred to a Bain coaxial circuit and maintained with the above anaesthetic mixture. At this stage, pre-operative antibiotics (5mg/kg

enrofloxacin), analgesia (0-05-0.1mg/kg buprenorphine) and warmed fluids (2ml of 0.9% saline) were administered subcutaneous (all drugs from Centaur Services, Somerset, UK). The left anterior chest wall was shaved in preparation for surgery. A modified 16G intravenous cannula with blunted stylet (Becton Dickinson, UK) was used as the endotracheal tube and passed into the trachea across the vocal cords under direct vision, with the trachea transillumination. The cannula was then connected to a Harvard Rodent Ventilator (DC1 55-0000 Small Animal Ventilator 683, Harvard Apparatus, MA, USA) and appropriate positioning of the endotracheal tube was confirmed by inspecting for periodic bilateral lung inflation. The rat was ventilated using volume-controlled ventilation at a cycle rate of 100 cycles per minute and a tidal volume of 2.0-2.5ml, giving a minute volume of 200-250ml/min. Maintenance anaesthesia was with a mixture of 2% isoflurane/98% oxygen. Adequacy of anaesthesia was monitored throughout the procedure using a combination of heart rate, respiratory rate and conscious reflexes.

Myocardial infarction surgery

Adult male Sprague-Dawley rats were subjected to left anterior descending (LAD) artery ligation to induce chronic myocardial infarction, as previously described (1, 2). Rats underwent left thoracotomy and the LAD artery was ligated under direct vision (**Supplementary Figure 1B**). Additional ligatures were placed around the LAD artery until visual confirmation of ischaemia (blanching and cyanosis of myocardium directly below ligature). MI surgery was performed blinded to treatment group.

***Ex vivo* optical mapping of transmembrane voltage (V_m) of Chronic MI hearts**

Chronically infarcted hearts were rapidly excised and perfused in a perspex optical mapping chamber (Cairn Research, Faversham, UK) following euthanasia by rapid cervical dislocation, consistent with methods described in Schedule 1 of the United Kingdom Animals (Scientific Procedures) Act 1986

(4). Before cervical dislocation, rats were anaesthetised with 5% isoflurane by placing them in an induction chamber. Explanted hearts were perfused with modified Krebs-Henseleit solution (in mmol/L: NaCl 118.5, $CaCl_2$ 1.85, KCl 4.5, Glucose 11.1, $NaHCO_3$ 25.0, $MgSO_4$ 2.5, NaH_2PO_4 1.4) gassed with 95% O_2/5% CO_2 at 37°C ± 0.5°C and pH 7.35 ± 0.05. Hearts were perfused at fixed coronary flow rates of 15ml/min during stabilization using a Peri-Star Pro peristaltic pump (WPI-Europe, Stevenage, UK). After a 15-minute stabilization period, hearts were perfused with a voltage-sensitive dye (25µl of 1mg/ml RH237 in DMSO; Invitrogen, Paisley, UK). Hearts were also perfused with 10µmol/L blebbistatin (Sigma-Aldrich, Gillingham, UK) to eliminate motion artefact (5, 6).

Cardiac optical action potentials were recorded using an optical mapping system, as previously described (7), and as shown in **Supplementary Figure 1C**. Hearts were illuminated using monochromatic LEDs, and excited at wavelength 530nm. Emitted light was focussed, and split with a dichroic mirror at 630 nm. The longer wavelength portion (>630nm) was passed through an emission filter (>660 nm) and focussed on a Hamamatsu 256 photo-diode array detector (16 x 16 square pixel array) (Cairn Research, Faversham, UK). Optical signals were recorded at a sampling rate of 1kHz with a spatial resolution of 1mm. The shorter wavelength (<630nm) light portion was focused onto a charge-coupled device (CCD) camera to obtain plain images of the heart in the mapping chamber (example in **Supplementary Figure 1C**). Signals were recorded at baseline during both sinus rhythm and during pacing from the left ventricular free wall (cycle length 200ms, 300bpm, stimulus 1mA) using a stimulator (MicroPace, Auckland, New Zealand). Optical imaging data were acquired and displayed using QRecord software (Cairn Research, Faversham, UK).

Optical mapping data were analysed using Optiq software (Cairn Research Ltd, Kent, UK), using analysis principles as previously described (8). Local activation times were assigned based on the

maximum first derivative of the fluorescent signal (dF/dt max) (9). Activation maps were generated, and local conduction velocities and vectors were derived, using MATLAB R2010a software (MathWorks, Massachusetts, USA), using methods previously described (8, 10, 11). The optical action potential rise time is defined as time between takeoff (maximum value of d^2F/dt^2) and the peak of the action potential. The action potential duration (APD) dispersion was the calculated as the standard deviation of APD values. The dispersion of conduction vector angle was quantified by determining the mean vector angle and then calculating the standard deviation of conduction vector angles from the mean vector. Optical mapping data pixels from the photo-diode array were matched to the plain image of the heart obtained with the CCD camera. Pixels within the scar area (white on the CCD image) were designated as infarct zone, and pixels within the viable myocardium (grey on the CCD image) were designated remote, non-infarcted myocardium. Pixels at the boundary of scar and myocardium were designated as infarct border zone pixels.

Arrhythmia Provocation Protocols

Hearts were subjected to programmed electrical stimulation (PES) protocols to measure vulnerability of hearts to re-entry arrhythmias. Hearts were paced from the left ventricle with a 20-beat drive train (cycle length 120ms, 500bpm, stimulus 1mA) followed by the addition of up to three extrastimuli. The vulnerability of hearts to PES-induced arrhythmias was quantified using a previously described Arrhythmia Inducibility Score for PES in rat hearts (12). Hearts were deemed positive for VT/VF if 6 or more ventricular beats were provoked by PES. If there were 15 or more VT/VF beats, this was classified as sustained. Each heart was assigned an Arrhythmia Inducibility Score from 0 to 6 (6 = sustained VT/VF with one extrastimulus, 5 = non-sustained VT/VF with one extrastimulus, 4 = sustained VT/VF with two extrastimuli, 3 = non-sustained VT/VF with two extrastimuli, 2 = sustained VT/VF with three extrastimuli, 1 = non-sustained VT/VF with three extrastimuli, 0 = no

arrhythmia induced). Arrhythmia provocation protocols were performed and analyzed blinded to the treatment group.

Infarct Size Analysis

Cryostat sections (10 µm) of flash frozen hearts were stained with Masson's trichrome (**Supplementary Figure 1D**). Digital images were acquired with high-resolution scanning of slides using an HP Scanjet G2710 scanner. To measure infarct size, planimetry analysis was performed offline using Adobe Photoshop CS3 v10.0. Using a previously validated planimetry protocol (13, 14), the endocardial and epicardial circumferences of the infarct were measured for each section, and the infarct size quantified as the proportion of the endocardial and epicardial circumferences bounded by the transmural infarct. The extent of infarction was measured by this method instead of measuring volumes as the latter may underestimate the original loss of myocardium because of the thinning of the infarct and the compensatory hypertrophy of remote non-infarcted myocardium (15).

Histomorphometric Analysis of the Infarct Border Zone

Masson's Trichrome-stained sections were used for histomorphometry to assess the heterogeneity of fibrosis at the infarct border zone (**Supplementary Figure 1E**). All analyses of images were performed blinded to treatment group. The infarct border zone (IBZ) was localized under light microscopy using a Nikon –Eclipse TE200 microscope. Digital images were acquired using a Nikon digital sight DS-Vi1 camera and NIS-Elements BR 3.2 software using a x20 zoom lens (**Supplementary Figure 2Bi**). Images were then analysed offline using ImageJ Software (NIH). Each image was converted into a RGB (red, green, blue) stack. The greatest contrast between fibrosis and surviving myocardium was observed in the Red colour image of each RGB stack, and this image was analyzed for each section. Thresholding and smoothing were then performed to accurately identify the islands of fibrosis and the lines of interface between fibrosis and surviving

myocardium. The threshold was set and recorded for each individual image by the operator. A smoothing factor of 10 provided the minimum filtering required to allow consistent removal of noise. (**Supplementary Figure 2Bii**). The total areas covered by fibrotic tissue and the lengths of interface between fibrotic tissue and surviving myocardium were quantified on ImageJ (**Supplementary Figure 2Bii**).

The degree of heterogeneity of fibrosis was then quantified using an Interface Complexity Ratio (ICR), defined as the ratio of the length of interface between fibrosis and surviving myocardium to the area of fibrosis in that microscopic field (**Supplementary Figure 2A**). As shown in the three hypothetical examples, infarct border zones with greater heterogeneity of fibrosis have greater ICRs, i.e. greater interface between fibrotic and myocardial tissue per unit area of fibrosis. The inter-observer and intra-observer coefficients of variation for this method were 12% and 10% respectively.

For the histomorphometric analyses, the entire ventricles were serially sectioned, and sections of 10μm thickness were selected for staining with Masson's Trichrome at 500μm intervals until the entire infarct was sampled from apex to base. For each 10μm section, the infarct border zone (IBZ), defined as the region of interface between scar (stained blue) and surviving myocardium (stained pink), was analysed. For each slice, microscopic IBZ images were recorded at intervals across the entire IBZ for offline histomorphometric analysis of scar heterogeneity, with 27 ± 5 IBZ microscopic fields analysed per slice. The Interface Complexity Ratios from multiple microscopic IBZ fields were averaged for each 10μm slice. The ICR values for the multiple sampled slices were averaged to give a single mean value and a single standard deviation value per heart. This method ensured the systematic sampling of the IBZ, which was consistent across all hearts.

Cx43 Immunohistochemistry

Sections were also immunolabeled for Cx43 as previously described (16, 17). Cryostat sections were incubated with a mouse anti-Cx43 primary antibody (Chemicon MAB 3067) then with a Cy3-conjugated goat anti-mouse secondary antibody (Chemicon) (**Supplementary Figure 1F**). Distribution of Cx43 was quantified using a previously-described Cx43 lateralisation score (17). Six immunolabeled slices were analysed for each heart. Analysis was performed blinded to treatment group.

Characterizing the effects of Rotigaptide on ventricular myocardium

In addition to the *in vivo* myocardial infarction protocols described above and in the main manuscript, we also performed a series of experiments to confirm the effects of rotigaptide on gap junction function in rat ventricular myocardium, by conducting immunoblotting and further *ex vivo* optical mapping studies.

To assess the effects of rotigaptide on Cx43 phosphorylation and expression in the context of acute ischaemia, 8 rat hearts were explanted and perfused *ex vivo* on a Langendorff apparatus. After stabilization, hearts were perfused with 50nM rotigaptide (n=4) or control perfusate (n=4) for 20 minutes. The left anterior descending artery was then ligated for 30 minutes as previously described (7). At the end of 30 minutes ischaemia, the ischaemic portions of the hearts were flash-frozen for quantitative immunoblotting of Cx43 as previously described (18). The primary antibody directed against Cx43 (MAB 3067 Chemicon International) at 1:500 dilution and an alkaline phosphatase conjugated anti-mouse secondary antibody (Pierce) at 1:2500 dilution were used for these experiments.

To determine the effects of rotigaptide on myocardial electrophysiological properties, optical mapping studies were performed in a metabolic stress model in 8 hearts. A global low-flow

ischaemia protocol (reduction of coronary flow rate to 33% of baseline with non-oxygenated perfusate) was used as the model of metabolic stress. After stabilization, hearts were treated with rotigaptide (n=3) or control perfusate (n=5) for 20 minutes. Hearts were then subjected to metabolic stress for 30 minutes before perfusion with oxygenated perfusate at normal coronary flow rates. Transmembrane voltage (Vm) transients were recorded every minute during sinus rhythm and ventricular pacing at a cycle length of 200ms. Optical mapping signals were analysed as described above.

Statistical analysis

Statistical analyses were performed using Prism 5.0 software (GraphPad Software, California, USA). Analysis of variance (ANOVA) tests were performed to compare mean values between multiple groups and post-hoc Tukey's test was subsequently used if ANOVA was significant. Student's T-tests were used to compare means between two groups. $P<0.05$ was considered significant. All values shown are mean ± S.E.M, unless otherwise stated.

Supplementary Results

Effects of rotigaptide on Cx43 phosphorylation status

Supplementary Figure 3A shows representative Cx43 immunoblots for ischaemic tissue from a control heart and a heart treated with rotigaptide. The three bands represent Cx43 protein with different degrees of phosphorylation at the carboxy-terminus (19). P0 is the dephosphorylated Cx43 isoform, phosphorylation at amino acid position S364 and/or S365 is involved in forming the P1 isoform, whilst phosphorylation at S325, S328 and/or S330 is necessary to form a P2 isoform. Rotigaptide was found to significantly reduce P0 and increase P1 and P2 in ischaemic tissue compared to control (**Supplementary Figure 3B & 3C**). The increase in P1 and P2 isoforms may be the mechanism by which rotigaptide attenuates conduction slowing in the metabolic stress below.

Effects of rotigaptide on conduction velocity

During acute metabolic stress, there was slowing of conduction over 30 minutes of metabolic stress, as shown by the crowding of isochrones in the activation maps in **Supplementary Figure 3D**. Metabolic stress resulted in a 41.1 ± 5.6 % slowing of conduction. Rotigaptide attenuated this conduction slowing in the context of metabolic stress, with a peak reduction in conduction velocity of 15.6 ± 4.6 % ($p<0.05$ vs. control), as shown in **Supplementary Figure 3E**. For both these groups, conduction velocity was restored to that of baseline levels after 10 minutes of perfusion with oxygenated perfusate at normal coronary flow rates. Taken together, the immunoblotting and optical mapping experiments provide evidence supporting previous observations that rotigaptide administration in the whole heart enhances gap junction coupling during acute ischaemia/metabolic stress.

Effect of rotigaptide on IBZ scar heterogeneity

As described in the main manuscript, rotigaptide significantly reduced the heterogeneity of fibrosis at the IBZ. These results were based on the analysis of multiple histological slices across the entire 3-dimensional infarct, as illustrated in **Supplementary Figure 4**. Although the mean ICR, which was calculated by averaging ICR values across multiple slices for each heart, was not different between the two groups, the dispersion of ICRs, calculated as the standard deviation of ICR for each heart, was significantly reduced in the rotigaptide group. The increased dispersion of ICR in the control group compared with the rotigaptide group, in the context of comparable mean ICRs, can be attributed to a greater range of IBZ morphologies throughout the entire infarct in the control hearts, with greater maximum ICR values in the control hearts. This means that the regions of maximum scar heterogeneity in the control hearts were more heterogeneous than the regions of maximum scar heterogeneity in the rotigaptide hearts, which had a smaller spread of ICR values closer to the mean. Additional histological images are presented in **Supplementary Figure 5** at two different magnifications (x4 and x20) to illustrate this finding, showing qualitatively that regions of maximum scar heterogeneity in the control hearts were more heterogeneous than the regions of maximum scar heterogeneity in the rotigaptide hearts

Supplementary References:

1. Lyon AR, Macleod KT, Zhang Y, et al. Loss of T-tubules and other changes to surface topography in ventricular myocytes from failing human and rat heart. Proc Natl Acad Sci USA 2009;106:6854–6859.

2. Lyon AR, Bannister ML, Collins T, et al. SERCA2a gene transfer decreases sarcoplasmic reticulum calcium leak and reduces ventricular arrhythmias in a model of chronic heart failure. Circ Arrhythm Electrophysiol 2011;4:362–372.

3. Haugan K, Marcussen N, Kjolbye A, Nielsen M, Hennan J, Petersen J. Treatment with the gap junction modifier rotigaptide (ZP123) reduces infarct size in rats with chronic myocardial infarction. J Cardiovasc Pharmacol 2006;47:236–242.

4. Parliament. Animals (Scientific Procedures) Act 1986. 1986.

5. Dou Y, Arlock P, Arner A. Blebbistatin specifically inhibits actin-myosin interaction in mouse cardiac muscle. Am J Physiol Cell Physiol 2007;293:C1148–53.

6. Fedorov V, Lozinsky I, Sosunov E, et al. Application of blebbistatin as an excitation-contraction uncoupler for electrophysiologic study of rat and rabbit hearts. Heart Rhythm 2007;4:619–626.

7. Ng FS, Shadi IT, Peters NS, Lyon AR. Selective heart rate reduction with ivabradine slows ischaemia-induced electrophysiological changes and reduces ischaemia-reperfusion-induced ventricular arrhythmias. J Mol Cell Cardiol 2013;59:67–75.

8. Laughner JI, Ng FS, Sulkin MS, Arthur RM, Efimov IR. Processing and analysis of cardiac optical mapping data obtained with potentiometric dyes. Am J Physiol Heart Circ Physiol 2012;303:H753–H765.

9. Efimov IR, Nikolski VP, Salama G. Optical imaging of the heart. Circ Res 2004;95:21–33.

10. Ng FS, Holzem KM, Koppel AC, et al. Adverse remodeling of the electrophysiological response to ischemia-reperfusion in human heart failure is associated with remodeling of metabolic gene expression. Circ Arrhythm Electrophysiol 2014;7:875–882.

11. Salama G, Kanai A, Efimov IR. Subthreshold stimulation of Purkinje fibers interrupts ventricular tachycardia in intact hearts. Experimental study with voltage-sensitive dyes and imaging techniques. Circ Res 1994;74:604–619.

12. Belichard P, Savard P, Cardinal R, et al. Markedly different effects on ventricular remodeling result in a decrease in inducibility of ventricular arrhythmias. J Am Coll Cardiol 1994;23:505–513.

13. Pfeffer J, Pfeffer M, Fletcher P, Braunwald E. Progressive ventricular remodeling in rat with myocardial infarction. Am J Physiol 1991;260:H1406–14.

14. Pfeffer M, Pfeffer J, Steinberg C, Finn P. Survival after an experimental myocardial infarction: beneficial effects of long-term therapy with captopril. Circulation 1985;72:406–412.

15. Pfeffer M, Pfeffer J, Fishbein M, et al. Myocardial infarct size and ventricular function in rats. Circ Res 1979;44:503–512.

16. Yao J-A, Hussain W, Patel P, Peters NS, Boyden PA, Wit AL. Remodeling of gap junctional channel function in epicardial border zone of healing canine infarcts. Circ Res 2003;92:437–443.

17. Patel PM, Plotnikov A, Kanagaratnam P, et al. Altering ventricular activation remodels gap junction distribution in canine heart. J Cardiovasc Electrophysiol 2001;12:570–577.

18. Hussain W, Patel PM, Chowdhury RA, et al. The Renin-Angiotensin system mediates the effects of stretch on conduction velocity, connexin43 expression, and redistribution in intact ventricle. J Cardiovasc Electrophysiol 2010;21:1276–1283.

19. Solan JL, Lampe PD. Key connexin 43 phosphorylation events regulate the gap junction life cycle. J Membr Biol 2007;217:35–41.

<u>Supplementary Figure 1:</u> Chronic myocardial infarction (MI) studies. (A) Study protocol (PES: Programmed electrical stimulation, IHC: immunohistochemistry). (B) Myocardial infarction surgery with solid black line indicating location of suture and dashed yellow lines indicating course of left anterior descending (LAD) artery. (C) Experimental set-up for optical mapping of transmembrane voltage in chronically-infarcted hearts. (D) Masson's Trichrome-stained biventricular slice for histomorphometry. (E) Complex pattern of scarring at infarct border zone with interdigitation of fibrosis (blue) with surviving myocardium (red). (F) Cx43 immunolabeling at the infarct border zone showing lateralisation of Cx43.

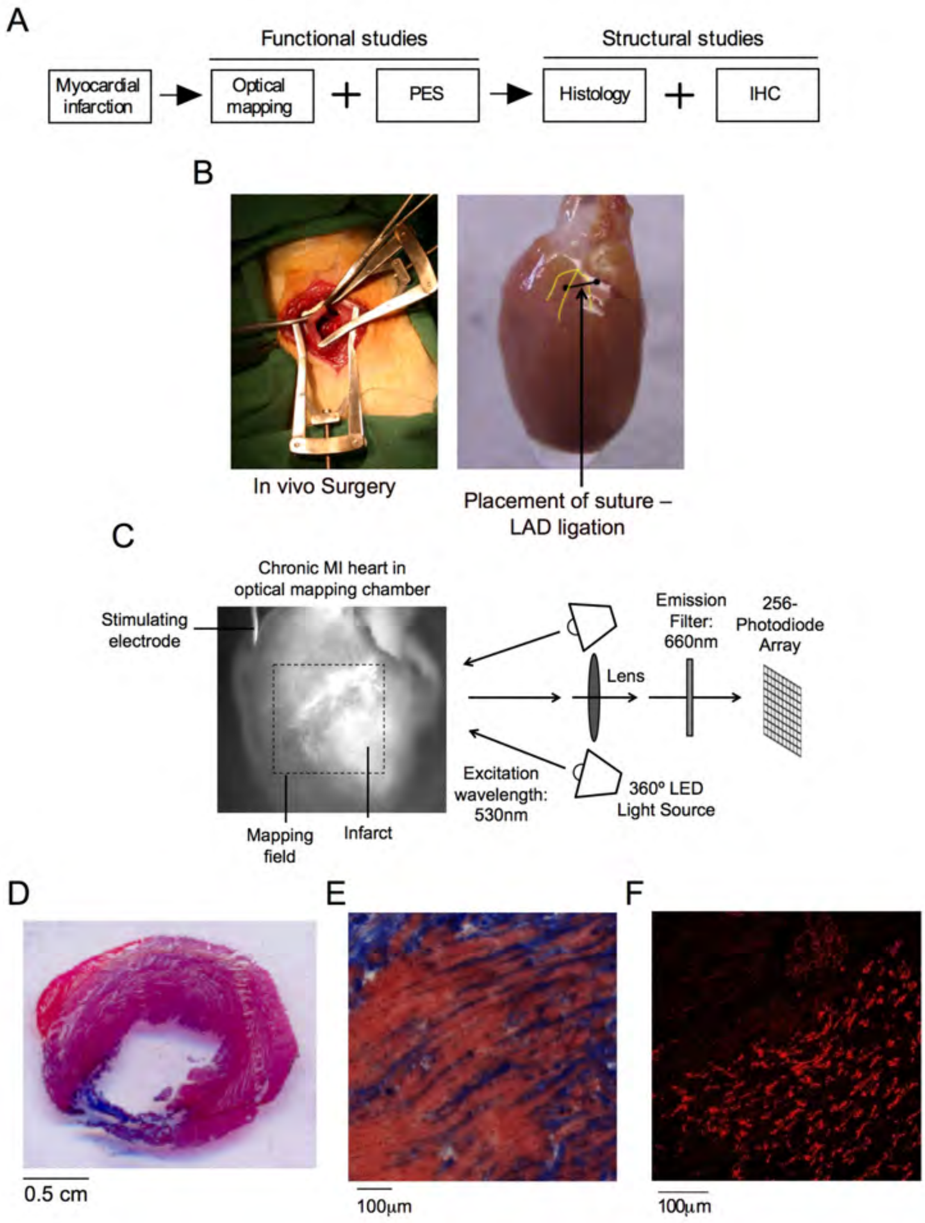

<u>Supplementary Figure 2:</u> Infarct Border Zone Histomorphometry. (A) The Interface Complexity Ratio (ICR) was used to quantify the heterogeneity of fibrosis. As shown by the three hypothetical examples of infarct border zone morphologies, complex heterogeneous scarring would produce high ratios, whereas homogeneous scarring would produce low ratios. (B) Processing of microscopic images to quantify ICR. (i) Images were taken with the x20 lens on the light microscope, focusing on the infarct border zone. (ii) Thresholding and smoothing were then performed to selectively identify islands/strands of fibrosis (highlighted in red). (iii) The perimeter (yellow lines) and area of these islands/strands of fibrosis were then computed using ImageJ, which were then used to calculate the ICR for that microscopic field.

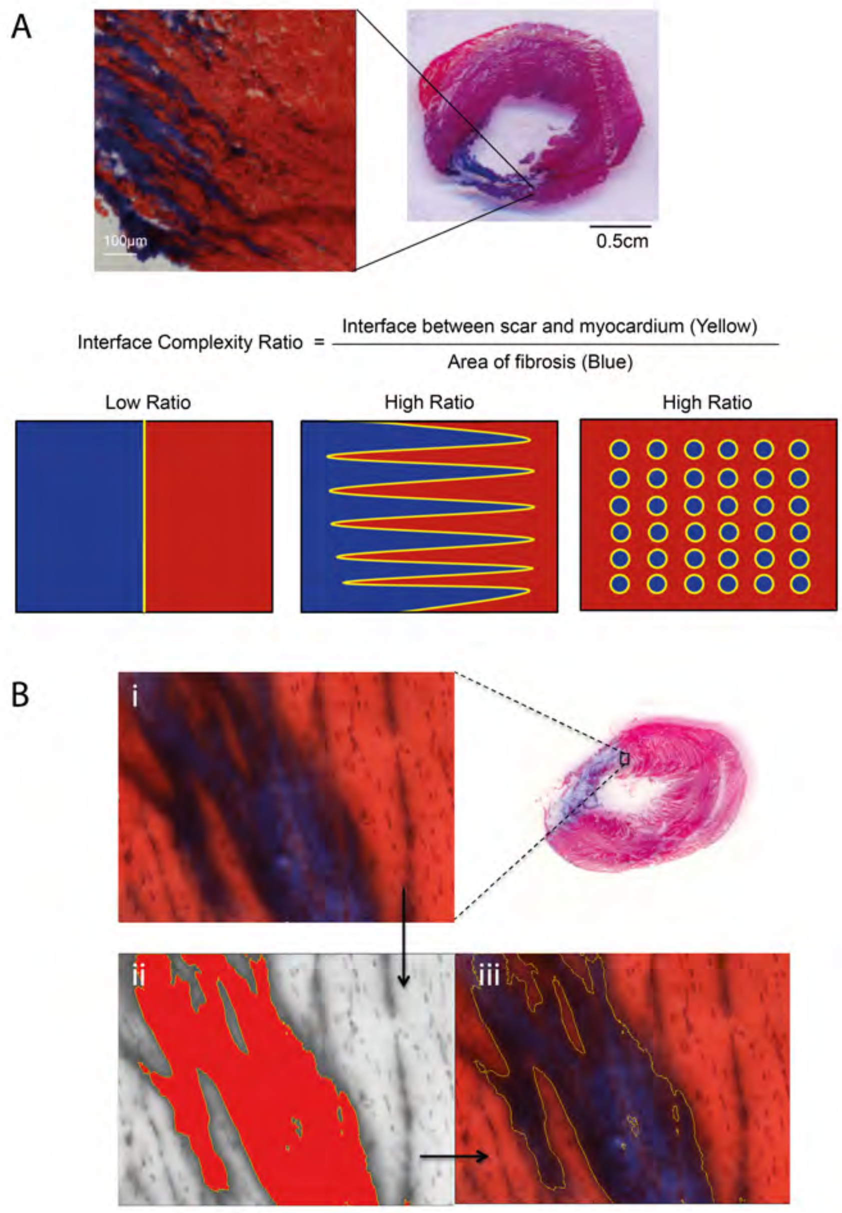

<u>Supplementary Figure 3:</u> Effects of rotigaptide on Cx43 phosphorylation status and conduction velocity. (A) Representative Cx43 immunoblots for ischaemic tissue from a heart treated with rotigaptide (ROT) and a control heart (CON). (B & C) Rotigaptide treatment increased the proportion of the phosphorylated Cx43 isoform (P2 and P1), and reduced the proportion of dephosphorylated Cx43 isoform (P0) (*p<0.05). (D) Isochronal maps at baseline and after 30 minutes of metabolic stress. (D & E) Rotigaptide treatment attenuated conduction slowing during acute metabolic stress.

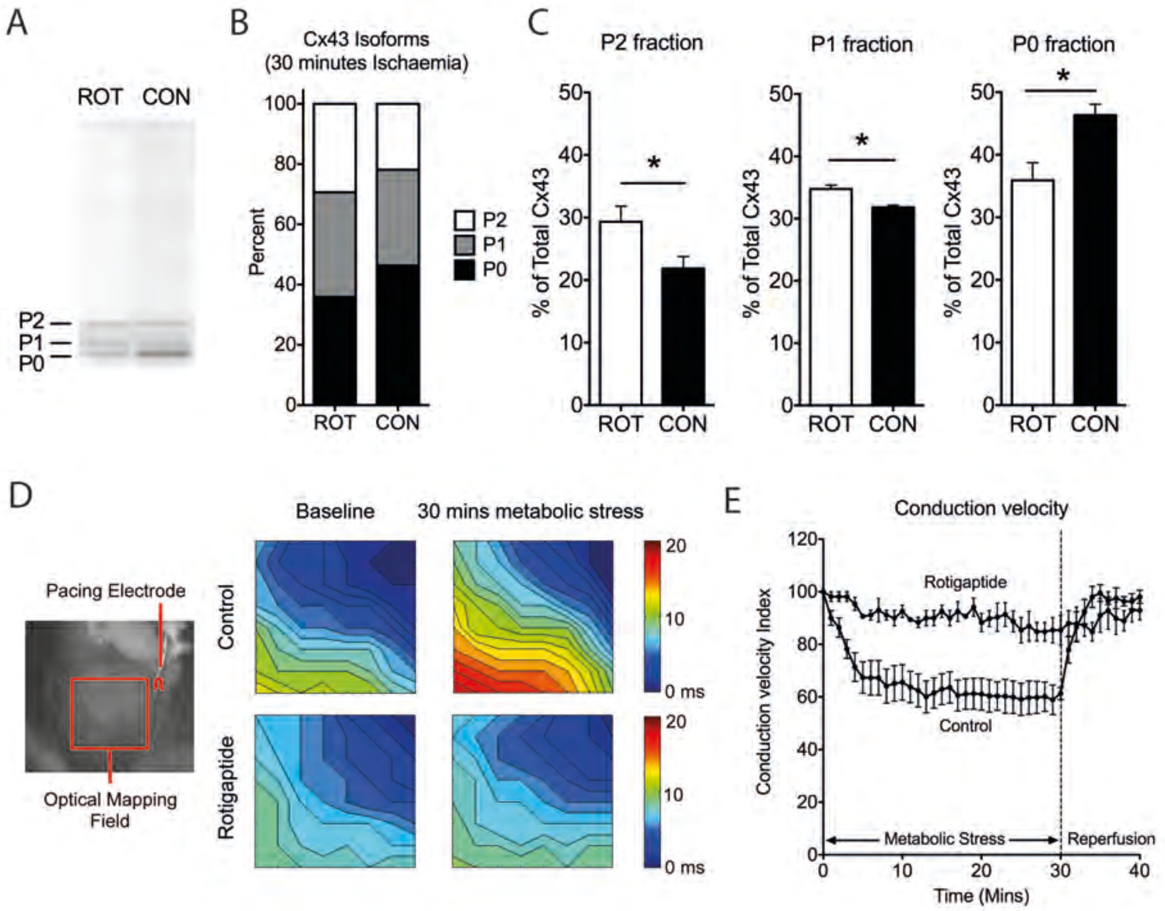

<u>Supplementary Figure 4:</u> Histological images of the IBZ from multiple slices across the 3-dimensional infarct. For each heart, 10µm slices were taken at 500µm intervals from across the entire infarct for staining with Masson's Trichrome for histomorphometry. Mean and standard deviations of ICRs therefore provided information across the entire 3-dimensional infarct. Eight images from the IBZ from eight consecutive slices from a control heart are shown below. Fibrosis is stained blue and surviving myocardium stained pink by Masson's Trichrome.

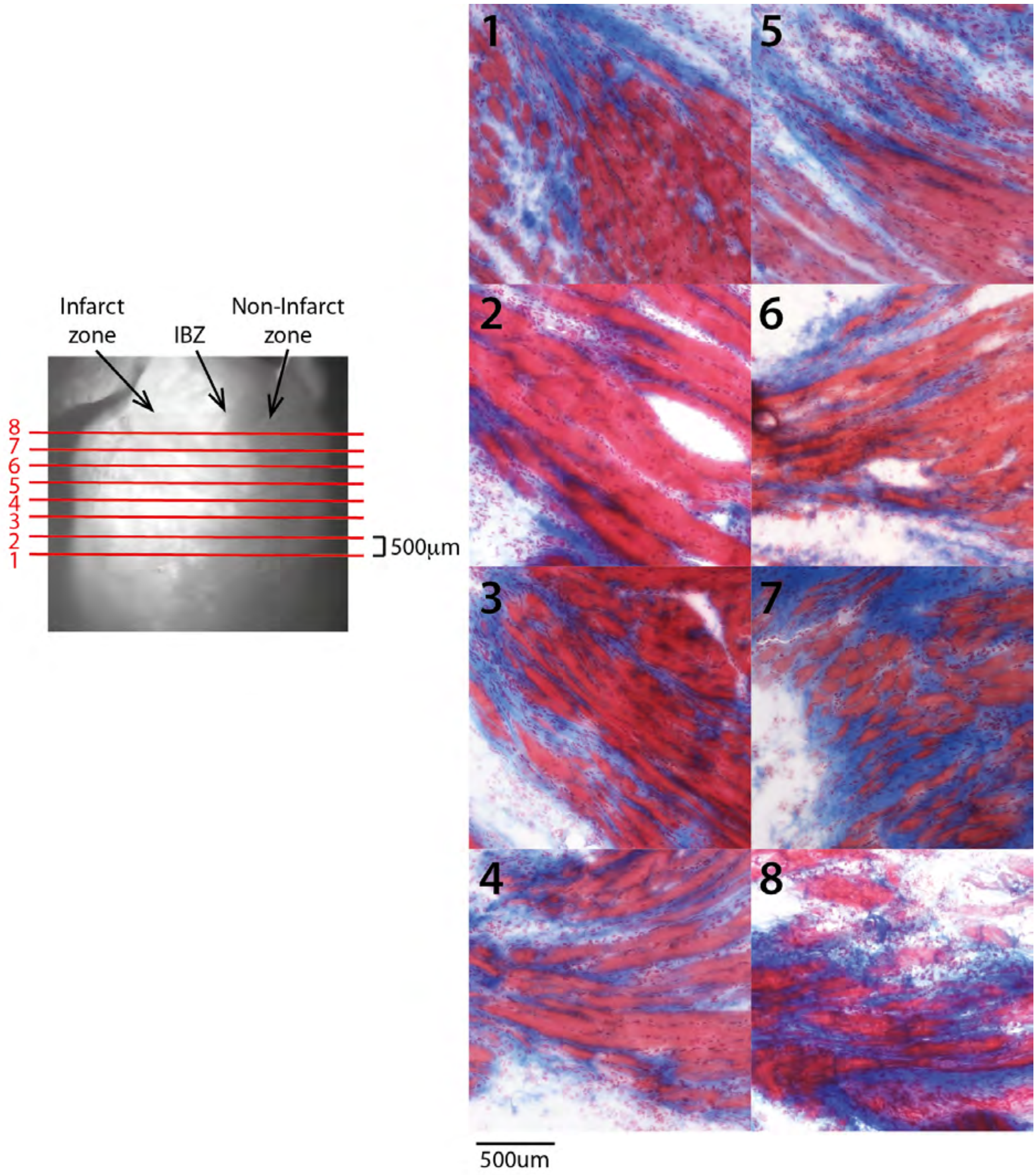

__Supplementary Figure 5: __**Reduction in heterogeneity of IBZ scarring in rotigaptide-treated hearts.** There was a reduction in the dispersion (standard deviation) of ICR for rotigaptide-treated hearts, which pointed towards more homogeneous patterns of IBZ scarring in rotigaptide hearts, whereas control hearts exhibited a greater range of IBZ scar morphologies within each heart. Images of IBZ at two microscopic magnifications (x20 and x4) illustrate this finding, showing qualitatively that regions of maximum scar heterogeneity in the control hearts were more heterogeneous than the regions of maximum scar heterogeneity in the rotigaptide hearts.

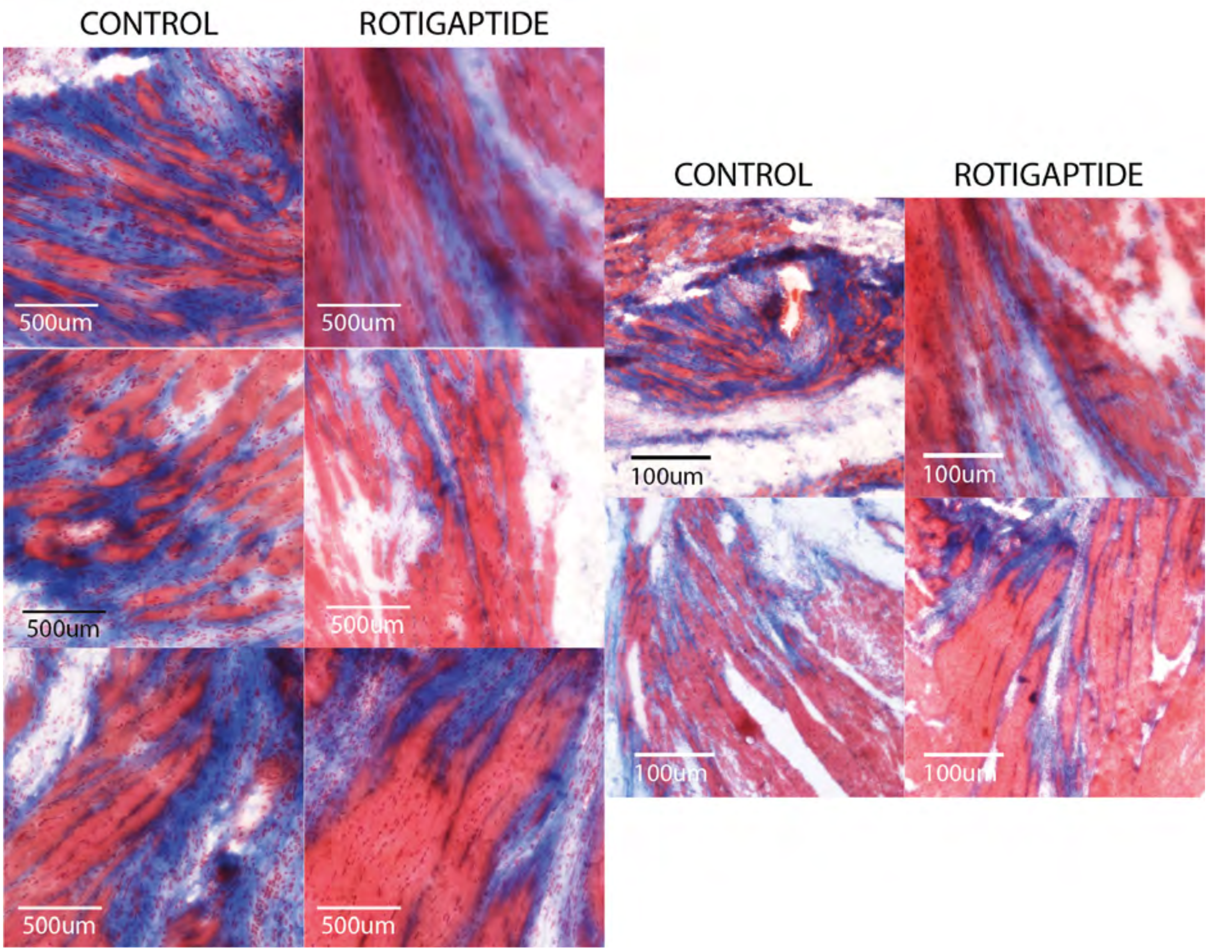

<u>Supplementary Figure 6:</u> Schematic of one proposed mechanism of effect of gap junction enhancement during acute MI on acute cell death and subsequent scar morphology. (A) During acute myocardial infarction, heterogeneous cell death occurs at the ischemic border, partly as a result of the natural closure of gap junction channels (red blocks: ischemic cells, blue blocks: non-ischemic cells at ischemic border zone). With rotigaptide, the maintenance of gap junction coupling allows the free exchange of both mediators of cell death (red arrows) and of cell survival (blue arrows) between cells at the ischemic border. (B) This free passage of mediators of cell death and rescue messengers leads to more homogeneous and less patchy cell death, and therefore a more homogeneous infarct scar.

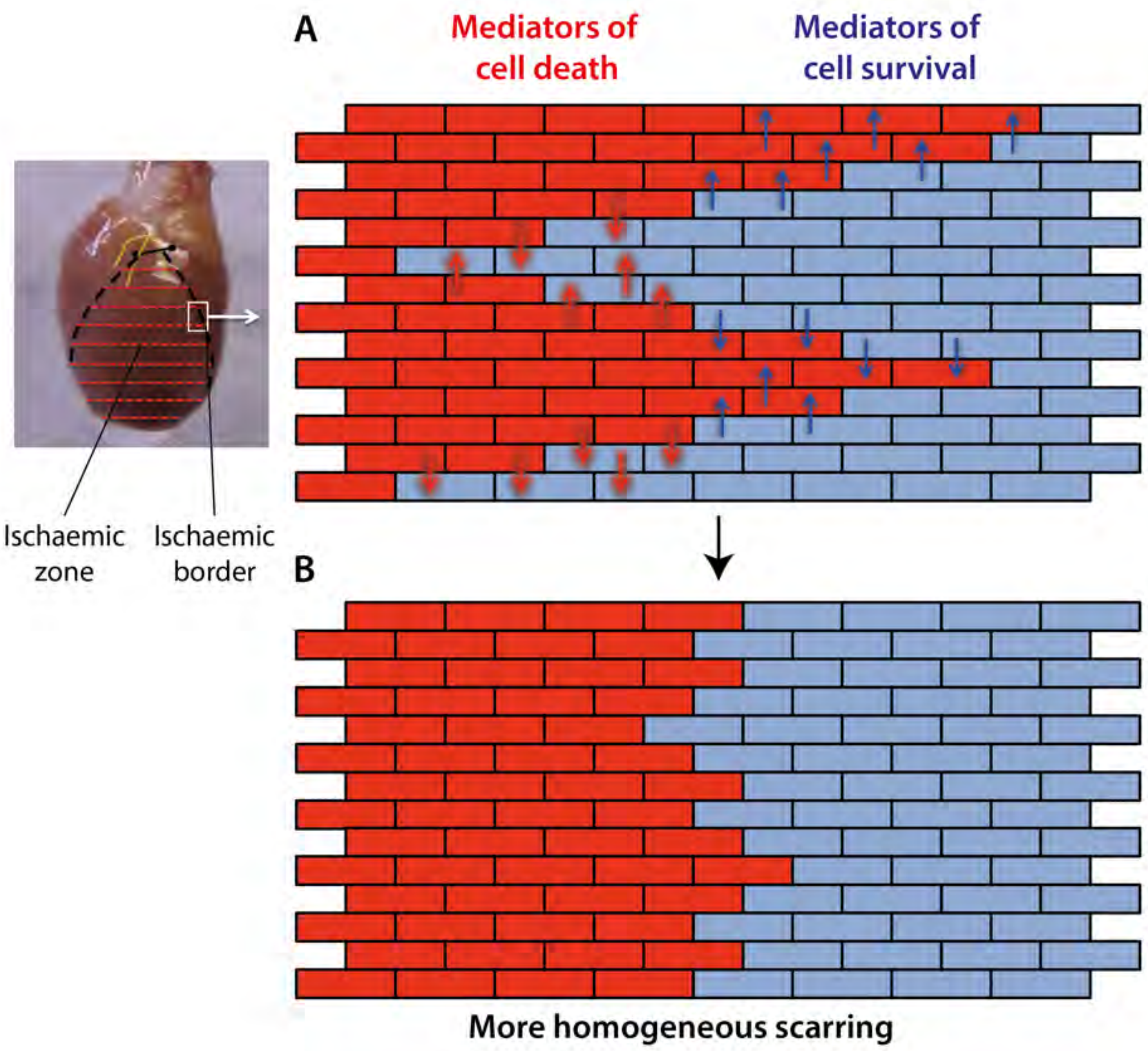

Supplement: Online Data [file mmc1.pdf]
